# Deaths Related to Chagas Disease and COVID-19 Co-Infection, Brazil, March–December 2020

**DOI:** 10.3201/eid2811.212158

**Published:** 2022-11

**Authors:** Francisco R. Martins-Melo, Marcia C. Castro, Antonio Luiz P. Ribeiro, Jorg Heukelbach, Guilherme L. Werneck

**Affiliations:** Federal Institute of Education, Science, and Technology of Ceará, Fortaleza, Brazil (F.R. Martins-Melo);; Harvard T.H. Chan School of Public Health, Boston, Massachusetts, USA (M.C. Castro);; Universidade Federal de Minas Gerais, Belo Horizonte, Brazil (A.L.P. Ribeiro);; Federal University of Ceará, Fortaleza (J. Heukelbach);; Federal University of Rio de Janeiro, Rio de Janeiro, Brazil (G.L. Werneck);; State University of Rio de Janeiro, Rio de Janeiro (G.L. Werneck)

**Keywords:** Chagas disease, COVID-19, coronavirus disease, SARS-CoV-2, severe acute respiratory syndrome coronavirus 2, coronaviruses, viruses, respiratory infections, American trypanosomiasis, Trypanosoma cruzi, parasites, protozoa, deaths, mortality rate, co-infection, zoonoses, Brazil

## Abstract

We analyzed epidemiologic characteristics and distribution of 492 deaths related to Chagas disease and coronavirus disease (COVID-19) co-infection in Brazil during March‒December 2020. Cumulative co-infected death rates were highest among advanced age groups, persons of Afro-Brazilian ethnicity and with low education levels, and geographically distributed mainly in major Chagas disease‒endemic areas.

Chagas disease, caused by the protozoan *Trypanosoma cruzi*, is a neglected public health problem in Latin America (*1*). It is the most common infectious cause of cardiomyopathy worldwide and for co-infections might play a role in clinical prognosis of COVID-19 patients (*2**,**3*). In Brazil, ≈1.4–3.4 million persons were estimated to be chronically infected with *T. cruzi* during 2015; 0.4–1.0 million of those persons had chronic Chagas heart disease (*4*).

On March 11, 2020, the World Health Organization declared COVID-19 a pandemic (*5*). In Brazil, a case of COVID-19 was detected on February 26, 2020, and a death from COVID-19 occurred on March 12, 2020 (*5*). COVID-19 vaccination campaigns started in late January 2021 (*6*). By September 18, 2022, there were >33.7 million confirmed cases and ≈685,000 deaths in Brazil (*7*). 

Spread of COVID-19 in Chagas disease‒endemic areas is a public health challenge because of advanced age of chronically infected patients and high occurrence of heart complications (*2*). This finding probably increases risk for severe forms and deaths from COVID-19 in co-infected patients (*5**,**8*). We assessed epidemiologic characteristics and distribution of deaths related to COVID-19 and Chagas disease co-infection in Brazil during March-December 2020.

## The Study

We conducted a nationwide analysis using mortality rate data for 2020 (preliminary records), obtained from the Brazilian Mortality Information System database (https://datasus.saude.gov.br/transferencia-de-arquivos) and extracted on September 4, 2021. We included all deaths reported from March 1‒December 31, 2020, in which Chagas disease (International Classification of Diseases, 10th revision [ICD-10], codes B57–57.5, K23.1, and K93.1) and COVID-19 (ICD-10 codes B34.2, U0.71 or U0.72) were mentioned on the same death certificate as underlying or contributing to death.

Available sociodemographic and clinical data included sex (male, female), age (<1–19, 20–29, 30–39, 40–49, 50–59, 60–69, 70–79, >80 years), education (years of study: none, 1–3, 4–7, 8–11, >12), ethnicity (White, Black/Afro-Brazilian, mixed/Pardo Brazilian, Asian, indigenous), marital status (single, married, divorced/separated, widowed, other), place of residence (regions, states, municipalities), date of death (epidemiologic week, month), place of death (hospital, other health establishment, home, public thoroughfare, others), and underlying/contributing causes of death. We calculated cumulative mortality rates per 100,000 inhabitants and rate ratios with 95% CIs stratified by sex, age group, place of residence, ethnicity, and educational level using population estimates from the Brazilian Institute of Geography and Statistics as the denominator. We assessed significant differences by χ^2^ test, performed analyses using Stata version 11.2 (StataCorp, https://www.stata.com), and created maps using ArcGIS version 9.3 (Esri, https://www.esri.com). Data were obtained anonymized, with no possibility of subject identification.

**Figure 1:**
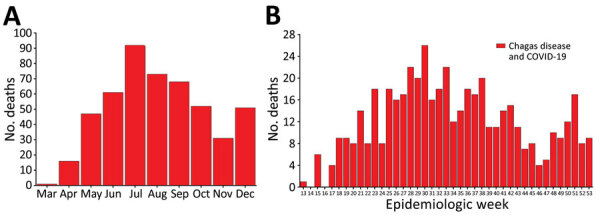
Number of deaths related to Chagas disease and COVID-19 co-infection, by month (A) and epidemiologic week (B) of death, Brazil, March–December 2020. Data shown are from the epidemiologic week of the first reported death related to Chagas disease and COVID-19 co-infection (March 26, 2020) to December 31, 2020 (epidemiologic weeks from 13 [March 22–28, 2020] to 53 [December 27, 2020–January 2, 2021; data available until December 31, 2020], according to the 2020 epidemiologic calendar (https://portalsinan.saude.gov.br/calendario-epidemiologico-2020). Red bars indicate the number of deaths related to Chagas and COVID-19 co-infection.

Of 1,337,730 deaths recorded in Brazil during March‒December 2020, we identified 492 deaths in which Chagas disease and COVID-19 were on the same death certificates (9.1% [492/5,395] of Chagas disease‒related deaths and 0.2% [492/222,121] of COVID-19‒related deaths for that period). The cumulative co-infected mortality rate was 0.23 (95% CI 0.21–0.25) deaths/100,000 inhabitants. COVID-19 was mentioned as the underlying cause in most co-infected deaths (88.2% [434/492]), of which 77.2% (335/492) were laboratory-confirmed COVID-19 deaths (B34.2 + U07.1). Chagas disease was the underlying cause in 7.7% (38/492) of co-infected deaths, with predominance of the chronic cardiac form (B57.2) (Table 1). The number of co-infected deaths peaked in July, in epidemiologic week 30 (July 19–25) (Figure 1), following the patterns of COVID-19 deaths during the 2020 pandemic time (Appendix Figures 1‒3).

**Table 1:** Underlying causes on death certificates that listed Chagas disease and COVID-19 co-infection, Brazil, March–December 2020*

Underlying causes of death (ICD-10 codes)	No. (%)
Coronavirus disease 19 – COVID-19 (B34.2, U07.1, U07.2)†	434 (88.2)
Coronavirus infection, unspecified site + COVID-19, virus identified (laboratory confirmed) (B34.2 + U07.1)	335 (68.1)
Coronavirus infection, unspecified site + COVID-19, virus not identified (clinically or epidemiologically diagnosed (B34.2 + U07.2)	52 (10.6)
Coronavirus infection, unspecified site (B34.2)	47 (9.6)
Chagas disease (B57, K23.1, K93.1)	38 (7.7)
Chagas disease (chronic) with heart involvement (B57.2)	27 (5.5)
Chagas disease (chronic) with digestive system involvement (B57.3)	6 (1.2)
Acute Chagas disease with heart involvement (B57.0)	3 (0.6)
Chagas disease (chronic) with nervous system involvement (B57.4)	2 (0.4)
Pneumonia (J12-J18)	3 (0.6)
Chronic obstructive pulmonary disease (J40-J44)	3 (0.6)
Diabetes mellitus (E10-E14)	2 (0.4)
Hypertensive diseases (I10-I15)	2 (0.4)
Other respiratory disorders (J98)	1 (0.2)
Infection due to other mycobacteria (A31)	1 (0.2)
Sepsis (A40-A41)	1 (0.2)
Secondary and unspecified malignant neoplasm of lymph nodes (C77)	1 (0.2)
Dementia (F00-F04)	1 (0.2)
Other disorders of brain (G93)	1 (0.2)
Appendicitis (K35-K37)	1 (0.2)
Paralytic ileus and intestinal obstruction without hernia (K56)	1 (0.2)
Cholelithiasis (K80)	1 (0.2)
Maternal infectious and parasitic diseases classifiable elsewhere but complicating pregnancy, childbirth and puerperium (O98)	1 (0.2)
Total	492 (100.0)

*COVID-19, coronavirus disease; ICD-10, International Statistical Classification of Diseases and Related Health Problems, 10th revision. †ICD-10 codes were based on the Brazilian Ministry of Health codification guidelines for COVID-19 (http://plataforma.saude.gov.br/cta-br-fic/codificacao-Covid-19.pdf), which recommends the standardized use of the ICD-10 code B34.2 (Coronavirus infection, unspecified site) for deaths of COVID-19 in Brazil, with the inclusion of pandemic marker codes U07.1 (COVID-19, identified virus) or U07.2 (COVID-19, unidentified virus, clinical or epidemiologic criteria), defined by the World Health Organization, next to code B34.2 in the same line of the death certificate.

Overall, co-infected deaths were predominant among men (51%), persons 70–79 years of age (37%), persons of mixed ethnicity (44.9%), married persons (44.5%), persons who had schooling (1–3 years of study) (33.2%), and persons who resided in the Southeast region (43.7%) and São Paulo state (27%). The mean (+SD) age at death was 73.9 (+12.2) years, median (range) age at death was 75.5 (30.7–104.4) years, and 87% of deaths occurred in hospitals (Table 2).

**Table 2:** Epidemiologic characteristics and cumulative mortality rates per 100,000 inhabitants related to Chagas disease and COVID-19 co-infection by population subgroups, Brazil, March–December 2020*

Characteristic	Deaths, no. (%)†	Cumulative mortality rate (95% CI)‡	Mortality rate ratio (95% CI)	p value
All co-infected deaths	492 (100.0)	0.23 (0.21–0.25)		
Sex				
M	251 (51.0)	0.24 (0.21–0.27)	1.09 (0.91–1.30)	0.345
F	241 (49.0)	0.22 (0.20–0.25)	Referent	
Age group, y				
30–39	6 (1.2)	0.02 (0.01–0.04)	Referent	
40–49	14 (2.8)	0.05 (0.03–0.08)	2.73 (1.05–7.10)	0.032
50–59	40 (8.1)	0.17 (0.12–0.23)	9.56 (4.05–22.54)	<0.001
60–69	104 (21.1)	0.62 (0.51–0.75)	35.46 (15.57–80.75)	<0.001
70–79	182 (37.0)	2.02 (1.74–2.33)	115.08 (51.03–259.53)	<0.001
≥80	146 (29.7)	3.29 (2.80–3.87)	187.56 (82.90–424.34)	<0.001
Region of residence				
North	7 (1.4)	0.04 (0.02–0.08)	Referent	
Northeast	97 (19.7)	0.17 (0.14–0.21)	4.51 (2.09–9.71)	<0.001
Southeast	215 (43.7)	0.24 (0.21–0.28)	6.44 (3.04–13.68)	<0.001
South	18 (3.7)	0.06 (0.04–0.09)	1.59 (0.66–3.81)	0.293
Central-West	155 (31.5)	0.94 (0.80–1.10)	25.05 (11.75–53.43)	<0.001
Ethnicity§				
White (Caucasian)	204 (43.0)	0.22 (0.19–0.25)	Referent	
Mixed race (Pardo Brazilians)	213 (44.9)	0.22 (0.19–0.22)	0.98 (0.81–1.19)	0.838
Black (Afro-Brazilian)	55 (11.6)	0.30 (0.23–0.39)	1.36 (1.01–1.83)	0.042
Asian	2 (0.4)	NA	NA	NA
Schooling, y				
None (illiteracy)	93 (25.3)	0.62 (0.50–0.76)	24.90 (11.55–53.67)	<0.001
1–3	122 (33.2)	0.68 (0.57–0.81)	27.34 (12.76–58.55)	<0.001
4–7	99 (26.9)	0.23 (0.19–0.27)	9.09 (4.22–19.56)	<0.001
8–11	47 (12.8)	0.07 (0.05–0.09)	2.64 (1.19–5.83)	0.013
≥12	7 (1.9)	0.02 (0.01–0.05)	Referent	
Marital status				
Single	60 (13.8)	0.07 (0.05–0.09)	0.19 (0.15–0.26)	<0.001
Married	194 (44.5)	0.34 (0.30–0.40)	Referent	
Divorced/separated	33 (7.6)	0.42 (0.30–0.59)	1.23 (0.85–1.77)	0.278
Widowed	136 (31.2)	1.69 (1.43–1.99)	4.91 (3.94–6.11)	<0.001
Other	13 (3.0)	NA	NA	NA
Place of occurrence of death				
Hospital	428 (87.0)	NA	NA	NA
Other health establishment	40 (8.1)	NA	NA	NA
Home	23 (4.7)	NA	NA	NA
Other	1 (0.2)	NA	NA	NA

*IQR, interquartile range; NA, not available. †Deaths with missing information are excluded: 18 for ethnicity, 124 for schooling, and 56 for marital status (not included in percentage calculations). ‡Deaths per 100,000 inhabitants. Population denominators used 2020 population estimates from the Brazilian Institute of Geography and Statistics (https://datasus.saude.gov.br/populacao-residente), except for ethnicity, schooling, and marital status. Population estimates for Brazil by ethnicity in 2020 were derived from the Continuous National Household Sample Survey (Continuous PNAD; https://sidra.ibge.gov.br/Tabela/6403), based on median estimates for each category (White, Black, and mixed [Pardo Brazilian]) in the continuous quarterly national surveys conducted in 2020. Population data on marital status in persons >10 years of age were obtained from the 2010 Brazilian Population Census (Brazilian Institute of Geography and Statistics, https://sidra.ibge.gov.br/tabela/1624). Population data on education for persons >10 years of age were extracted from the National Household Sample Survey (PNAD; https://sidra.ibge.gov.br/tabela/272) by using estimates for 2015 (most recent year with schooling estimates stratified by year of study >1 to 15 years). §No measures were calculated for persons of Asian ethnicity because of lack of population denominator information from 2020 Continuous PNAD. There was no record of co-infected death in the indigenous ethnic group.

**Figure 2:**
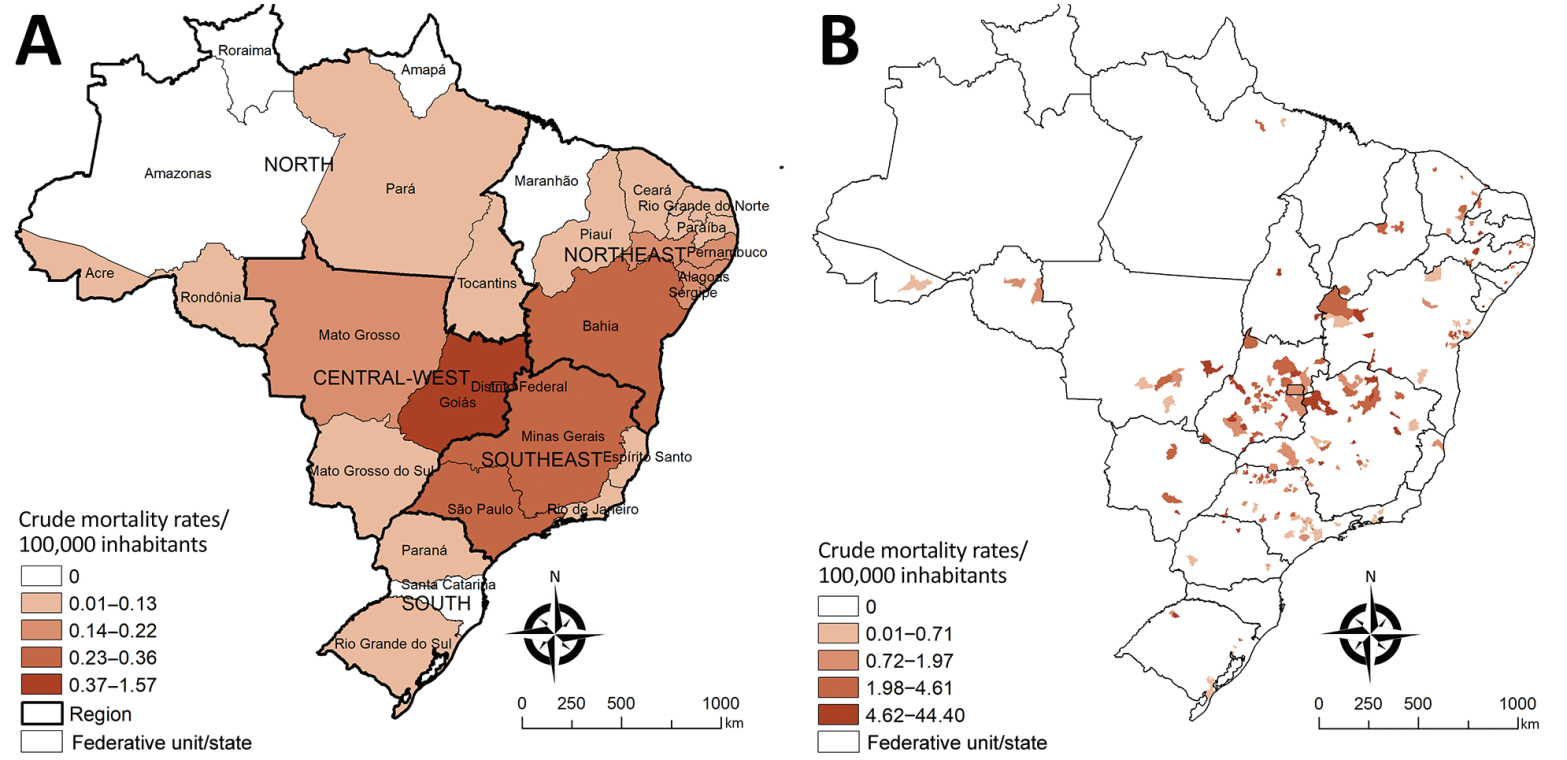
Spatial distribution of cumulative mortality rates per 100,000 inhabitants related to Chagas disease and COVID-19 co-infection by geographic units of residence, Brazil, March–December 2020. A) State-level crude rates. B) Municipality-level crude rates. Shading indicates levels of death. Data were mapped by using ArcGIS software version 9.3 (Esri, https://www.esri.com). In 2020, Brazil was divided into 5 regions (South, Southeast, Central-West, North, and Northeast), 27 Federative Units (26 states and 1 Federal District), and 5,570 municipalities.

Cumulative mortality rates were higher for men than for women, but not significantly. Highest age-specific mortality rates were found for older age groups, the maximum in persons >80 years of age (3.29/100,000 inhabitants). Persons of Afro-Brazilian ethnicity had higher mortality rates than did White persons. Mortality rates were higher among persons who had low levels of education (none and 1‒3 years of study) than persons who had advanced education. The Central-West region had the highest regional mortality rate, followed by Southeast and Northeast regions (Table 2). Federal District (1.57 deaths/100,000 inhabitants), Goiás (1.38 deaths/100,000 inhabitants), and Bahia (0.36 deaths/100,000 inhabitants) had the highest state-level mortality rates (Figure 2, panel A; Appendix Table).

A total of 4.1% (231/5,570) of municipalities in 22 of the 27 states of Brazil recorded >1 co-infected deaths during 2020. Cumulative mortality rates were 0.04–44.40 deaths/100,000 inhabitants among municipalities in Brazil that recorded >1 co-infected death. Municipalities that had high co-infected death rates were found mainly in the central region of Brazil (Goiás, Minas Gerais, São Paulo, Bahia, and the Federal District) (Figure 2, panel B).

## Conclusions

We found higher death rates for Chagas disease and COVID-19 co-infection among older persons, persons who had Afro-Brazilian ethnicity, persons with low education levels, and persons lived in an area to which Chagas disease was previously endemic. The high co-infection mortality rate for older age groups is consistent with patterns of deaths for both infections in Brazil during 2020 because the highest age-specific death rates for the diseases were for these subgroup populations (*9**–**11*). Consistent with other reports for both infections, we found that the higher death rates found for persons of Black ethnicity and with low educational levels indicate social and structural inequities and health disparities in determination of severe illness and death related to Chagas disease and COVID-19 in Brazil (*9**,**11**,**12*).

The areas of Brazil that had the highest mortality rates were major disease-endemic areas for vector transmission in the past in the Central-West, Southeast, and Northeast regions (*4**,**9**,**13*). The extensive spread of COVID-19 in Brazil during 2020, including Chagas disease-endemic areas, caused substantial geographic overlap between the infections, increasing the risk of chronic Chagas disease patients, principally those with cardiac involvement, contracting SARS-CoV-2 infection (*2**,**5*). The high mortality rate for the Federal District when compared with other states, and the high number of co-infected deaths observed in state capitals of Brazil, such as Brasília, São Paulo, Goiânia, and Salvador, reflect urbanization of Chagas disease because of intense migratory movement from rural areas to urban areas in Brazil during the past 3 decades (*9**,**14*).

Our study’s limitations were mainly related to coverage and quality of secondary mortality rate data (*9**,**13*). Brazilian Mortality Information System data for 2020 are preliminary and might not represent all deaths for 2020 because it is subject to corrections, especially underlying causes of death. Even if minimal, frequencies might change after definitive consolidation (*15*). Other potential limitations are misclassification or underreporting and delays in reporting of COVID-19 deaths, especially in places where healthcare services were under stress because of the large COVID-19 caseload (*6*).

It is likely that a large number of patients who have chronic Chagas disease, especially the undetermined form, are not given a diagnosis in Brazil. Therefore, there might be more deaths of patients who have both infections than reported in this study. Schooling, ethnicity, and marital status included a considerable proportion of incomplete/unknown data, and these findings should be interpreted with caution.

Our findings show marked sociodemographic and geographic variations in deaths related to Chagas disease and COVID-19 co-infection in Brazil during 2020, occurring mainly in residents of Chagas disease‒endemic areas and disproportionately affecting susceptible population groups. The real effect of death from co-infection might be underestimated in Brazil. Efforts must be made to ensure a high COVID-19 vaccination coverage, improve access to healthcare services, and provide adequate clinical management for co-infected patients especially in patients who have chronic Chagas disease.

Appendix Additional information on deaths related to Chagas disease and COVID-19 co-infection, Brazil, March–December 2020.
