# Supplementary material for: Deaths Related to Chagas Disease and COVID-19 Co-Infection, Brazil, March–December 2020
Source: Emerg Infect Dis. 2022 Nov;28(11):2285–9. doi: 10.3201/eid2811.212158 (PMC9622242; doi:10.3201/eid2811.212158)
Supplement: Appendix — Additional information on deaths related to Chagas disease and COVID-19 co-infection, Brazil, March–December 2020. [file 21-2158-Techapp-s1.pdf]

Article DOI: https://doi.org/10.3201/eid2811.212158

Deaths Related to Chagas Disease and COVID-19 Co-Infection, Brazil, March–December 2020

Appendix

Appendix Table. Absolute number of deaths and cumulative crude mortality rates per 100,000 inhabitants related to Chagas disease and COVID-19 co-infection, by states of residence, Brazil, March–December 2020*

		Deaths		Cumulative mortality
Region	State	No.	%	rates (95% CI)†
North	Rondônia	2	0.4	0.11 (0.03–0.41)
	Acre	1	0.2	0.11 (0.02–0.63)
	Amazonas‡	NA	NA	NA
	Roraima‡	NA	NA	NA
	Pará	2	0.4	0.02 (0.01–0.08)
	Amapá‡	NA	NA	NA
	Tocantins	2	0.4	0.13 (0.03–0.46)
Northeast	Maranhão‡	NA	NA	NA
	Piauí	4	0.8	0.12 (0.05–0.31)
	Ceará	8	1.6	0.09 (0.04–0.17)
	Rio Grande do Norte	1	0.2	0.03 (<0.01–0.16)
	Paraíba	3	0.6	0.07 (0.03–0.22)
	Pernambuco	17	3.5	0.18 (0.11–0.28)
	Alagoas	5	1.0	0.15 (0.06–0.35)
	Sergipe	5	1.0	0.22 (0.09–0.50)
	Bahia	54	11.0	0.36 (0.28–0.47)
Southeast	Minas Gerais	70	14.2	0.33 (0.26–0.42)
	Espirito Santo	1	0.2	0.02 (<0.01–0.14)
	Rio de Janeiro	11	2.2	0.06 (0.04–0.11)
	São Paulo	133	27.0	0.29 (0.24–0.34)
South	Paraná	11	2.2	0.10 (0.05–0.17)
	Santa Catarina‡	NA	NA	NA
	Rio Grande do Sul	7	1.4	0.06 (0.03–0.13)
Central-West	Mato Grosso do Sul	3	0.6	0.11 (0.04–0.31)
	Mato Grosso	6	1.2	0.17 (0.08–0.37)
	Goiás	98	19.9	1.38 (1.13–1.68)
	Distrito Federal	48	9.8	1.57 (1.19–2.08)
Brazil		492	100.0	0.23 (0.21–0.25)

*COVID-19, coronavirus disease; NA. not available.
†Deaths per 100,000 inhabitants. Population denominator used 2020 population estimates from the Brazilian Institute of Geography and Statistics (IBGE; https://datasus.saude.gov.br/populacao-residente/).
‡States of Brazil that did not report deaths caused by co-infection during the study period.

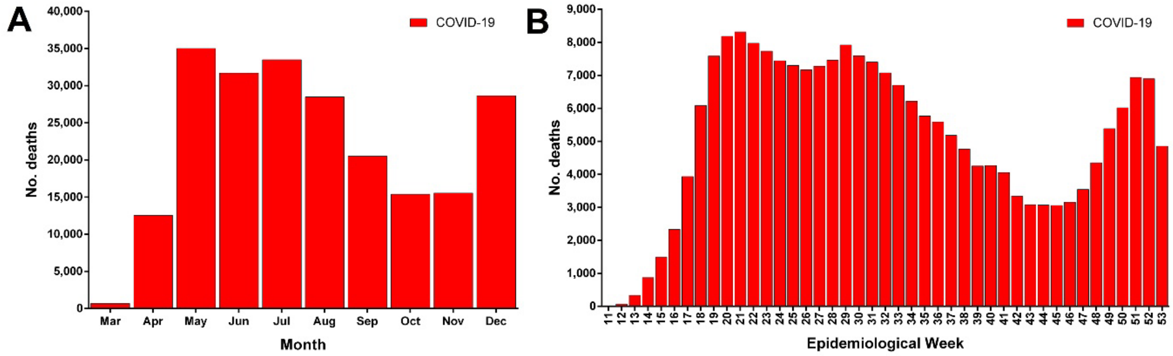

Appendix Figure 1. Number of coronavirus disease (COVID-19) deaths by A) month and B) epidemiologic week of death, Brazil, March–December 2020. Data shown from the epidemiologic week of the first documented death due to COVID-19 in Brazil (March 12, 2020) to December 31, 2020 (epidemiologic weeks from 11th [March 8–14, 2020] to 53th [December 27, 2020–January 2, 2021; data available until December 31, 2020], according to the 2020 epidemiologic calendar; http://portalsinan.saude.gov.br/calendario-epidemiologico-2020). Red bars indicate number of COVID-19 deaths.

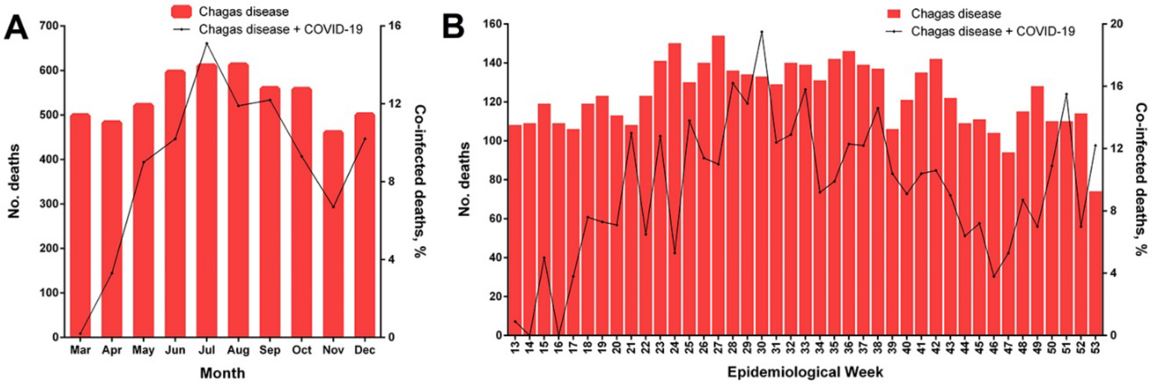

Appendix Figure 2. Number of Chagas disease-related deaths and proportion of deaths related to Chagas disease and COVID-19 co-infection by A) month and B) epidemiologic week of death, Brazil, March–December 2020. Data shown from the epidemiologic week of the first reported death related to Chagas disease and COVID-19 co-infection (March 26, 2020) to December 31, 2020 (epidemiologic weeks from 13th [March 22–28, 2020] to 53rd [December 27, 2020–January 2, 2021; data available until December 31, 2020], according to the 2020 epidemiologic calendar; http://portalsinan.saude.gov.br/calendario-epidemiologico-2020). Red bars indicate number of Chagas disease-related deaths (y-axis left scale). Black line indicates proportion of deaths related to Chagas disease and COVID-19 co-infection in relation to the total number of Chagas disease-related deaths (y-axis right scale). COVID-19, coronavirus disease.

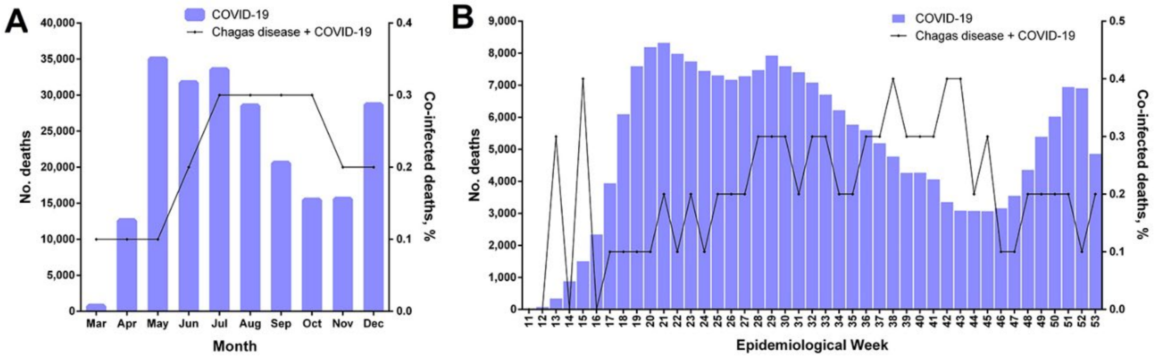

Appendix Figure 3. Number of COVID-19 deaths and proportion of deaths related to Chagas disease and COVID-19 co-infection by A) month and B) epidemiologic week of death, Brazil, March–December 2020. Data shown from the epidemiologic week of the first documented death due to COVID-19 in Brazil (March 12, 2020) to December 31, 2020 (epidemiologic weeks from 11th [March 8–14, 2020] to 53rd [December 27, 2020-January 2, 2021; data available until December 31, 2020], according to the 2020 epidemiologic calendar; http://portalsinan.saude.gov.br/calendario-epidemiologico-2020). Blue bars indicate number of COVID-19 deaths (left y-axis). Black line indicates proportion of deaths related to Chagas disease and COVID-19 co-infection in relation to the total number of COVID-19 deaths (right y-axis). COVID-19, coronavirus disease.
